# Changes in climate extremes, fresh water availability and vulnerability to food insecurity projected at 1.5°C and 2°C global warming with a higher-resolution global climate model

**DOI:** 10.1098/rsta.2016.0452

**Published:** 2018-04-02

**Authors:** Richard A. Betts, Lorenzo Alfieri, Catherine Bradshaw, John Caesar, Luc Feyen, Pierre Friedlingstein, Laila Gohar, Aristeidis Koutroulis, Kirsty Lewis, Catherine Morfopoulos, Lamprini Papadimitriou, Katy J. Richardson, Ioannis Tsanis, Klaus Wyser

**Affiliations:** 1 College of Life and Environmental Sciences, University of Exeter, Exeter EX4 4PS, UK; 2 Met Office Hadley Centre, FitzRoy Road, Exeter EX1 3PB, UK; 3 European Commission -- Joint Research Centre, 21027 Ispra, Italy; 4 College of Engineering, Mathematics and Physical Sciences, University of Exeter, Exeter EX4 4QE, UK; 5 School of Environmental Engineering, Technical University of Crete—TUC, Chania 73100, Greece; 6 Cranfield Water Science Institute, Cranfield University, Cranfield MK43 0AL, UK; 7 Rossby Centre, SMHI, 601 76 Norrköping, Sweden

**Keywords:** 1.5°C, Paris Agreement, 2°C, global climate impacts, water resources, terrestrial ecosystems

## Abstract

We projected changes in weather extremes, hydrological impacts and vulnerability to food insecurity at global warming of 1.5°C and 2°C relative to pre-industrial, using a new global atmospheric general circulation model HadGEM3A-GA3.0 driven by patterns of sea-surface temperatures and sea ice from selected members of the 5th Coupled Model Intercomparison Project (CMIP5) ensemble, forced with the RCP8.5 concentration scenario. To provide more detailed representations of climate processes and impacts, the spatial resolution was N216 (approx. 60 km grid length in mid-latitudes), a higher resolution than the CMIP5 models. We used a set of impacts-relevant indices and a global land surface model to examine the projected changes in weather extremes and their implications for freshwater availability and vulnerability to food insecurity. Uncertainties in regional climate responses are assessed, examining ranges of outcomes in impacts to inform risk assessments. Despite some degree of inconsistency between components of the study due to the need to correct for systematic biases in some aspects, the outcomes from different ensemble members could be compared for several different indicators. The projections for weather extremes indices and biophysical impacts quantities support expectations that the magnitude of change is generally larger for 2°C global warming than 1.5°C. Hot extremes become even hotter, with increases being more intense than seen in CMIP5 projections. Precipitation-related extremes show more geographical variation with some increases and some decreases in both heavy precipitation and drought. There are substantial regional uncertainties in hydrological impacts at local scales due to different climate models producing different outcomes. Nevertheless, hydrological impacts generally point towards wetter conditions on average, with increased mean river flows, longer heavy rainfall events, particularly in South and East Asia with the most extreme projections suggesting more than a doubling of flows in the Ganges at 2°C global warming. Some areas are projected to experience shorter meteorological drought events and less severe low flows, although longer droughts and/or decreases in low flows are projected in many other areas, particularly southern Africa and South America. Flows in the Amazon are projected to decline by up to 25%. Increases in either heavy rainfall or drought events imply increased vulnerability to food insecurity, but if global warming is limited to 1.5°C, this vulnerability is projected to remain smaller than at 2°C global warming in approximately 76% of developing countries. At 2°C, four countries are projected to reach unprecedented levels of vulnerability to food insecurity.

This article is part of the theme issue ‘The Paris Agreement: understanding the physical and social challenges for a warming world of 1.5°C above pre-industrial levels’.

## 1. Introduction

The majority of climate-change impacts assessments have tended to be framed in terms of future time horizons, e.g. impacts by the middle or end of the twenty-first century [1,2]. However, with international climate policy now largely focused on limiting warming to specific levels of global mean temperature such as 2°C [3] or 1.5°C [4], policy-relevant climate impacts assessments increasingly need to be framed in terms of such warming levels.

There are two major research questions concerning the impacts of climate change at 1.5°C and 2°C global warming, which are relevant to both mitigation and adaptation policy areas.

(i) How much larger are the impacts at 2°C compared to 1.5°C? This is the primary question arising from the Paris Agreement [4] and is relevant to mitigation policy, informing judgements and actions on holding the global temperature rise to ‘well below 2°C’ and ‘pursuing efforts to limit the temperature increase to 1.5°C’.
(ii) What regional climate conditions and related hydrological and ecological conditions could occur at a particular level of global warming, such as 2°C? This is relevant to adaptation policy and planning—exploring the possible outcomes for these levels of warming will help facilitate adaptation and improved resilience to account for a 1.5°C or 2°C world. It is recognized that many adaptation decisions require information on timing of specific impacts or risks, but nevertheless, framing regional impacts assessments in terms of associated global warming levels (GWLs) may help provide context of the levels of climate change that may be avoidable or unavoidable (and hence require adaptation).

A combination of the above questions is also relevant—how does the range of outcomes at 2°C compare to that at 1.5°C? This is also relevant to adaptation policy, as it can inform assessment on whether to adapt to potential impacts at 2°C or just 1.5°C. Putting in place adaptation measures to deal with potential impacts at 1.5°C and then increasing these to deal with 2°C later may be more expensive and difficult than adapting to potential risks at 2°C at the outset. On the other hand, because adaptation actions may themselves have consequences, unnecessary over-adaptation may have undesirable effects which it may be preferable to avoid or at least delay until absolutely necessary.

Both questions require an appropriate assessment of uncertainty. There are considerable uncertainties in projections of regional climate change, with different climate models projecting regional climate changes that can differ in magnitude or even, in the case of precipitation and impacts quantities strongly related to this, differ in sign [5,6]. This may have important implications for regional impacts at specific levels of global warming. A common approach to exploring and presenting such uncertainties is to examine the ensemble mean and the level of consensus among the ensemble members on the sign of the change. While this can often be useful in informing an assessment of the level of confidence in future projections, it may not always be sufficient to fully inform decisions. Risk assessment approaches require consideration of a range of possible risks, not just the most likely. This paper explores a range of regional climate states and related impacts that occur at global warming of 2°C, and a range of differences with warming limited to 1.5°C.

We examine the implications of our new climate projections by applying some commonly used indices of climate extremes, and a further index quantifying relative vulnerability to food insecurity which combines climate extremes indices with information on a range of factors representing sensitivity and adaptability of food systems to climate hazards. We also use the climate projections to drive a global land surface model to simulate changes in run-off as an indicator of freshwater availability. We assess whether regional extremes are projected to increase or decrease at 2°C global warming, and whether the consequent impact on drought and vulnerability to food insecurity become greater or smaller. We also assess whether these changes are reduced by limiting global warming to 1.5°C. We explore some of the uncertainties in these projections, and, in particular, examine whether the use of ensemble-mean projections is a useful simple guide to impacts projections or whether this can lead to a misleading impression for some impacts. Regarding vulnerability to food insecurity, we consider the impacts of global warming at 1.5°C and 2°C alongside socio-economic influences that affect the sensitivity to climate change. We also consider our climate-change impacts results in comparison with other studies using older, lower-resolution climate projections.

A large number of previous studies have assessed potential impacts of future climate change using the 5th Coupled Model Intercomparison Project (CMIP5) ensemble or subsets of this [7], and some have framed this in terms of impacts at global warming of 1.5°C and/or 2°C [8,9]. We also base our study on a subset of CMIP5 projections, but use a new, higher-resolution atmosphere model to provide greater spatial detail and improved representation of atmospheric processes.

## 2. Methods and models

### (a) Global climate simulations at 1.5°C and 2°C global warming

There are a number of ways in which 1.5°C or 2°C global warming can be defined—one could be the long-term climate state following a stabilization of warming at that level, another could be the state over a shorter period around the time of first reaching that level. Here we choose the second definition, which is what is seen first and hence needs to be adapted to. There are also a number of methods with which such changes can be assessed [10]. We take the opportunity of availability of a new set of higher-resolutions transient climate and impacts simulations, and use a time-sampling methodology [10] to assess global-scale impacts at these resolutions for the first time.

Rather than using the original CMIP5 ensemble as in previous studies, the aim is to allow for an improved representation of atmospheric and land surface processes including extremes by using higher spatial resolution [11].

HadGEM3 (Hadley Centre Global Environment Model version 3) is a configuration of the UK Met Office Unified Model (MetUM) which has been developed for use for both climate research and weather prediction applications. It is the result of converging the development of the Met Office's weather and climate global atmospheric model components so that, where possible, atmospheric processes are modelled or parametrized seamlessly across spatial resolutions and timescales.

The high-resolution simulations were performed using the HadGEM3A Global Atmosphere (GA) 3.0 model [12–14] at a resolution of N216 (0.556° of latitude by 0.833° of longitude with gridboxes of approx. 60 km length in mid-latitudes). This is the atmospheric component of the HadGEM3-GC2 coupled climate model [15,16], which is part of the HadGEM3 family of climate models [12]. This represents the third generation of HadGEM configurations, leading on from the HadGEM2 family of climate model configurations [13] which was used for CMIP5. Key improvements over the previous model, HadGEM2, include increased vertical levels in the atmosphere (85 compared to 38) and substantial changes to the model dynamics (ENDGame) [17]. This version of the HadGEM3 model lies in the transition from CMIP5 to CMIP6 versions. The Met Office is currently operationally running the coupled HadGEM3-GC2 model at N216 resolution for seasonal and decadal forecasting and clear benefits are emerging from this use at higher resolution [18,19].

We ran the model using only its atmosphere and land components, with time-varying sea-surface temperatures (SSTs) and sea-ice concentrations (SICs) prescribed as input quantities. This approach was taken for two reasons: (i) to provide a rapid first analysis of the implications of the higher resolution for projections of climate extremes and impacts—an atmosphere-only simulation requires considerably less computing time than a coupled ocean–atmosphere general circulation model (GCM); (ii) to allow us to explore, to some degree, uncertainties in regional climate changes by using SSTs and SICs from different climate models. To explore these uncertainties in the regional impacts of climate change, we carried out six HadGEM3 atmospheric simulations driven by time-varying SSTs and SICs from a subset of projections from the CMIP5 with the RCP8.5 scenario. The assumption here is that SSTs and SICs provide a substantial influence on regional patterns of climate change over land, so using a range of SST and SIC patterns in a single atmosphere model goes some way towards representing the range of regional climate changes that would arise in a set of different coupled ocean–atmosphere GCMs. This approach will not capture the full range of uncertainty affecting regional climate changes over land, because it still relies on one atmosphere model and one land surface scheme, so responses to radiative forcing that depend mainly on atmospheric process or land-atmosphere interactions will still be constrained by the behaviour of that single model. Nevertheless, we consider that our experimental design avoids the reliance on one single realization of climate and hence allows some of the uncertainties in regional climate-change impacts to be illustrated and explored.

The SSTs and SICs were taken from a subset of the CMIP5 transient projections performed with the RCP8.5 scenario from 1979 to 2100—the CMIP5 members were selected as representative of a range of outcomes for future climate change, including high and low climate sensitivity, different biases in baseline precipitation climatology, and different global patterns of precipitation change. Specific levels of global warming such as 1.5°C or 2°C were defined on the basis of the global mean temperature in the original CMIP5 projections. The time of reaching a specific level of global warming, therefore, varied between ensemble members. The CMIP5 SSTs were not bias-corrected, which means that the results here may be sensitive to systematic errors arising from biases in the present-day SST patterns.

Atmospheric greenhouse gas concentrations were prescribed from the standard RCP8.5 concentration scenario. Aerosol concentrations were calculated within the model, with aerosol emissions prescribed again from the standard RCP8.5 scenario. This means that the greenhouse gas and aerosol concentrations, and hence radiative forcing, were the same in all ensemble members at any given date. Since specific levels of global warming such as 1.5°C or 2°C were reached at different times in the different ensemble members, according to the SST forcings used, any given level of global warming could be associated with different radiative forcings in different ensemble members. In any given ensemble member at any specific level of global warming, the CO_2_ concentration and SSTs were the same as in the driving CMIP5 model at that GWL. Land cover was fixed in this simulation—there was no dynamic vegetation nor any time-dependent anthropogenic land use change.

Some comparison of the higher-resolution atmospheric simulations with the original CMIP5 simulations, is provided by Wyser *et al.* [20].

### (b) Temperature and precipitation extremes: the ClimPACT indices

To quantify changes in weather extremes projected in our climate simulations, we calculated a number of indices designed to be relevant to sector-specific impacts using an established methodology, ClimPACT [21] (table 1)

**Table 1.** ClimPACT weather extremes indices.

ID	definition	units	sector of relevance
TXx	annual maximum daily maximum temperature	°C	health, agriculture and food security
TX90p	percentage of days above the 90th percentile of daily maximum temperature in the 1981–2010 average	%	health, agriculture and food security, water resources and hydrology
CDD	maximum number of consecutive days with precipitation less than 1 mm	days	health, agriculture and food security, water resources and hydrology
RX5day	maximum consecutive 5 day precipitation	mm	health, agriculture and food security, water resources and hydrology

### (c) Food security: the Hunger and Climate Vulnerability Index

To assess implications of climate change for vulnerability to food insecurity, we used an adaptation of the Hunger and Climate Vulnerability Index (HCVI) [22]. The HCVI was developed by the United Nations World Food Programme to provide a country-level assessment of vulnerability to food insecurity as a result of climate-related events. We used a new iteration of the HCVI which makes use of gridded climate model projections to understand the impact of climate change on vulnerability to food insecurity, and the benefits that adaptation can bring via scenarios of adaptation investment [23]. This iteration of the HCVI only considers in-country production of food and does not account for food trade. For this reason, the HCVI is only calculated for 122 developing and least-developed countries (defined here as countries not in the OECD or EU which can be resolved by the scale of the climate model; i.e. larger than 500 km^2^).

The index provides quantification at the national level across the globe of the scale and direction of impact of climate change on food insecurity. As such, it aims to provide the following: (i) information to help policy-makers understand the level of challenge to global food security that climate change presents; (ii) information on the geography of the impacts and help to evaluate the relative benefits of mitigation and adaptation responses.

The index is not intended to be a detailed planning tool, but aims to help planners evaluate the nature of the top-level threat to food insecurity that climate change presents, thereby supporting prioritization of effort.

The HCVI consists of three equally weighted components: exposure to climate-related hazards, sensitivity of national agricultural production to climate-related hazards, and adaptive capacity—a measure of a country's ability to cope with climate-related food shocks. The sensitivity and adaptive capacity components are based on data from the World Bank, World Resources Institute, UN Food and Agriculture Organization, UN Development Programme and UN Population Fund [22]. The exposure component comprised proxies for the average length of flood and drought events calculated with daily precipitation data [23] (table 2). These proxies were chosen above other possible metrics as they were required to replace self-reported instances of flood and drought events used in the original HCVI, which correlate with undernutrition data at the country-level [23]. The proxies were therefore masked to only include data where a significant proportion of people live and grow crops before aggregating to country level and combining to comprise a measure of exposure [23]; nevertheless, it is recognized that precipitation data alone may not always be adequate for representing flood and drought events, so the current method is regarded as preliminary.

**Table 2.** Proxies for flood and drought events used in the HCVI.

extreme weather event	description of proxy
average length of flood events	number of days in which the cumulative daily rainfall excess is positive, compared with the 95th percentile in the 1981–2010 average
average length of drought events	number of days in which the cumulative daily rainfall deficit is positive, compared with the 20th percentile in the 1981–2010 average

The impacts of projected climate change, therefore, act through changes in these quantities. In the current version of the HCVI, climate-change impacts on other quantities such as crop yield are not considered. Socio-economic factors affecting sensitivity and adaptive capacity are fixed at present-day conditions.

**Figure 1.**
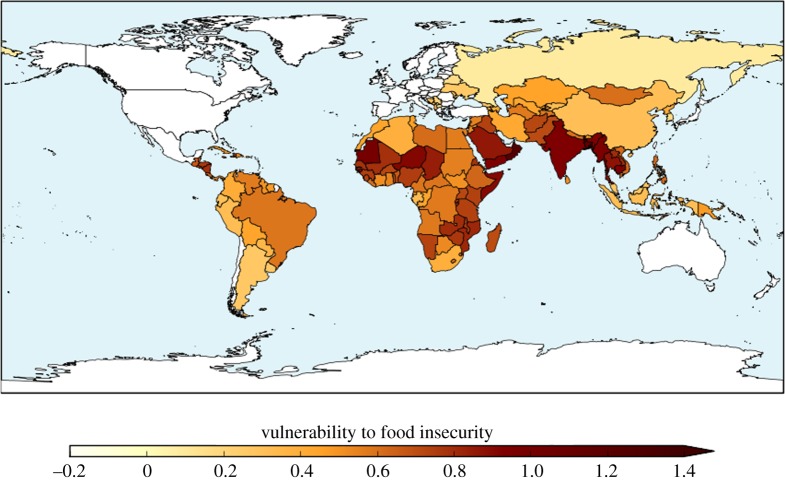
Hunger and Climate Vulnerability Index for 1981–2010 climate (ensemble mean across the bias-corrected HadGEM3 ensemble).

The ensemble-mean baseline HCVI calculated with the high-resolution bias-corrected HadGEM3 ensemble is shown in figure 1. The spatial pattern is compatible with HCVI values calculated using reanalysis data at the CMIP5 grid-scale resolution [23]; the most vulnerable regions are sub-Saharan Africa and South Asia. This higher-resolution climate data enables inclusion of additional countries which were not resolved in the lower-resolution CMIP5 data.

In the present study, processing errors in the input data for one ensemble member, the HadGEM2-ES-driven member, caused the results to be invalid. Results for this member for the HCVI are, therefore, not presented here.

### (d) Freshwater resources: run-off

Impacts on freshwater were assessed with a version of the JULES land surface model [24,25], a coupled ecosystem–hydrology–surface exchange model which simulates land-atmosphere fluxes of water, energy and carbon in an internally consistent way, typically applied at global scales. Variants of JULES form the land surface scheme of Met Office Hadley Centre Earth System Models [26,27] and have been used to assess impacts of climate change on global terrestrial ecosystems and hydrology [28–30] within such models. JULES can also be used outside of the Earth System Model (ESM), driven by meteorological outputs of other ESMs to assess impacts of a wider range of climate projections [6,8]. Here we use a new, higher-resolution configuration of JULES on a global grid of 0.5° resolution [31].

It has been noted that hydrological impacts models driven by climate-change projections from climate models tend to give more severe drying than simulated in the climate models themselves [32–34]. This is largely attributed to the inclusion of plant stomatal closure in response to elevated CO_2_ in the climate model land surface schemes, which generally reduces evapotranspiration relative to climate projections without this process and hence further increases run-off/streamflow or ameliorates decreases [34]. This process is often omitted from standard hydrological models. Plant physiological responses to CO_2_ are included in the JULES model, so our projections of changes in run-off here do account for this process.

We used each HadGEM3 simulation to drive JULES to simulate changes in run-off due to the effects of climate change and CO_2_ rise on precipitation, evaporation and transpiration. We analysed 30 year periods centred around the year of crossing GWLs of 1.5°C and 2°C relative to pre-industrial. We examined changes in both mean flows and low flows (defined as the flows for the lowest 10% of time).

### (e) Correcting biases in climate model output and implications for defining levels of global warming

The ClimPACT extreme weather indices, HCVI and JULES run-off simulations were all performed using outputs from the higher-resolution HadGEM3 projections described in §2a. However, there were some differences in how these data were applied, with different approaches to the treatment of systematic biases in the climate model output. For the ClimPACT analysis, it was considered important to assess changes in the raw climate model output, because this directly represents the behaviour of the model itself. The main focus was on the changes relative to the present-day baseline climate, defined as 1981–2010, with absolute values in either the baseline or the GWLs of 1.5°C and 2°C being only of secondary interest. For the HCVI and JULES run-off analyses, however, it was considered important to correct for systematic biases in the climate model output, because these can lead to unrealistic representations of the key quantities in the present-day simulation [35]. A bias-correction methodology was, therefore, applied for these two parts of the analysis, whereby the model output was adjusted to make it consistent with an observed climatology [36]. We used a multi-segment statistical bias-correction methodology for precipitation [37], and a modification of this for other variables [37].

This difference in approach led to inconsistencies in the definitions of the dates of GWLs in the two parts of the study. In the extremes analysis using raw model output, the dates of passing GWLs were defined on the basis of the global mean temperatures in the driving CMIP5 models relative to those models' simulations of global mean temperature in 1870–1899 (table 3). However, in the HCVI and JULES analyses which used bias-corrected data, it was considered more appropriate for the GWLs to be defined using the warming in the observational dataset up to present-day plus model-projected warming thereafter (table 4). While this does lead to inconsistent definitions of dates of the GWLs for applications of the climate model output with and without bias correction, the focus here is on the level of warming relative to pre-industrial rather than the timing of this warming. Therefore, priority is given to an accurate quantification of GWLs in all parts of the study, at the expense of inconsistencies in the dates of these warming levels. The inconsistency between the dates of the GWLs ranged from 2 to 9 years depending on the model and warming level. This inconsistency would have consequences if these results were applied to time-dependent impacts and adaptation assessments, but that is not the case here so this concern does not apply. However, one issue is that the time-dependent nature of the aerosol forcing means that the spatial pattern of regional climate responses varies over time, so this will lead to some degree of inconsistency between the analysis of the ClimPACT extremes and the HCVI and JULES impacts projections.

**Table 3.** Time of reaching GWLs of 1.5°C and 2°C in the raw output from the HadGEM3 climate simulations, driven by different sets of CMIP5 sea-surface temperatures. The dates are the centre year of a 20-year period for which the climate data are applied to the calculation of the ClimPACT indices.

driving SSTs	1.5°C	2.0°C
IPSL-CM5A-LR	2015	2030
GFDL-ESM2M	2040	2055
HadGEM2-ES	2027	2039
IPSL-CM5A-MR	2020	2034
MIROC-ESM-CHEM	2023	2035
ACCESS1–0	2034	2046

**Table 4.** Time of reaching GWLs of 1.5°C and 2°C in each bias-corrected output from the HadGEM3 climate simulations, driven by different sets of CMIP5 sea-surface temperatures. The dates are the centre year of a 20 year period for which the climate data is applied to the HCVI calculation and JULES simulations.

driving SSTs	1.5°C	2.0°C
IPSL-CM5A-LR	2024	2035
GFDL-ESM2M	2036	2051
HadGEM2-ES	2019	2033
IPSL-CM5A-MR	2023	2036
MIROC-ESM-CHEM	2020	2032
ACCESS1-0	2026	2040

## 3. Results

For a world at 2°C global warming, we present a range of outcomes to provide insight into the level of agreement between models for a particular projected change, and hence an indication of potential robustness of the projected changes for informing adaptation. We then make a comparison of impacts at global warming 1.5°C to investigate the level of impact that would be avoided by limiting global warming to different levels. Bearing in mind the uncertainty in regional climate outcomes, we address this in a number of ways. For individual realizations, we compare the impacts at different warming levels to see if they are systematically smaller at 1.5°C, even if the sign of the change is uncertain. We also compare the range of outcomes at different GWLs, to see if the regional-scale uncertainty itself increases with global warming.

### (a) Climate-change impacts at 2°C global warming

**Figure 2.**
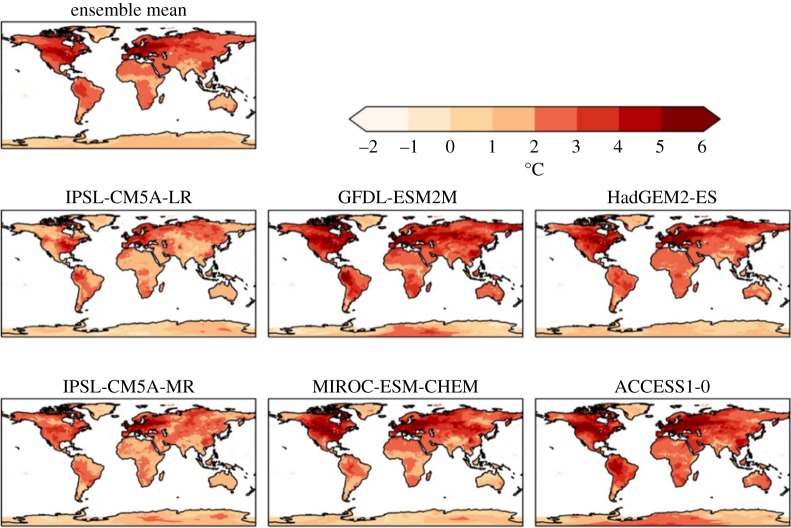
Simulated changes in annual daily maximum temperature relative to 1981–2010 at 2°C global warming, for individual HadGEM3 simulations driven by SSTs and SICs from different members of the CMIP5 ensemble, and the ensemble mean. The labels above each panel identify the driving CMIP5 model (or ensemble mean).

For 2°C global warming, the ensemble-mean increase in annual daily maximum temperature was above 2°C for most of the land surface, with the exception of the Indian subcontinent, most of Australia and Antarctica (figure 2). The increase was higher still in many regions; most of North America, much of China and north Asia, northwestern South America and all of Europe. In the northern and eastern USA and much of northern and western Europe, the annual daily maximum temperature increased by over 4°C for 2°C global warming. The global mean TXx increased by more than 2°C in all ensemble members (table 5), so the maximum temperature warming more than the global annual mean is a consistent result across all projections here, as found in previous studies with other models [9] (table 5).

**Table 5.** Global mean changes at 2°C global warming compared to present day for individual ensemble members, for the ClimPACT indices, the flood and drought proxies used as input to the HCVI calculations, and percentage change in mean precipitation (Pmean), mean run-off (Rmean) and low run-off (Rlow).

	IPSL-CM5A-LR	GFDL-ESM2M	HadGEM2-ES	IPSL-CM5A-MR	MIRC-ESM-CHEM	ACCESS1-0	ensemble mean
TXx (°C)	2.1	2.8	2.5	2.9	2.4	2.8	2.6
TX90p (% time)	20.1	24.3	24.9	29.0	23.5	27.9	25.0
CDD	−3.0	0.9	−3.4	−5.7	−2.0	−5.5	−2.9
RX5day (mm)	3.5	5.4	6.9	6.8	6.0	6.7	5.9
drought proxy	0.76	0.89	n.a.	0.38	0.38	0.66	0.61
flood proxy	0.83	0.82	n.a.	0.75	0.73	0.78	0.78
Pmean (%)	2.1	3.4	5.0	3.0	5.3	2.9	4.0
Rmean (%)	2.4	6.5	8.1	4.4	8.6	4.9	5.8
Rlow (%)	−2.0	3.8	11.2	8.0	9.4	5.1	5.9

The different ensemble members give somewhat different results at regional scales, although there is a strong consensus on the temperature extremes examined here becoming warmer. In the simulations driven by SSTs and SICs from the two IPSL CMIP5 models, most of the global land surface sees an increase in annual daily maximum temperature which is similar to the global annual mean temperature increase. In the IPSL-driven simulations, increases in TXx substantially larger than the GWL are confined to the eastern USA, Europe and part of northeast Asia. By contrast, the GFDL-driven simulation shows much of the global land surface seeing increases in annual daily maximum temperature larger than the global mean warming. Much of the mid-latitudes experience an increase in TXx of over 4°C. The very largest increases of 5°C or more are seen in central North America, Europe and northwestern Asia. Similar results are seen in the MIROC and ACCESS models.

**Figure 3.**
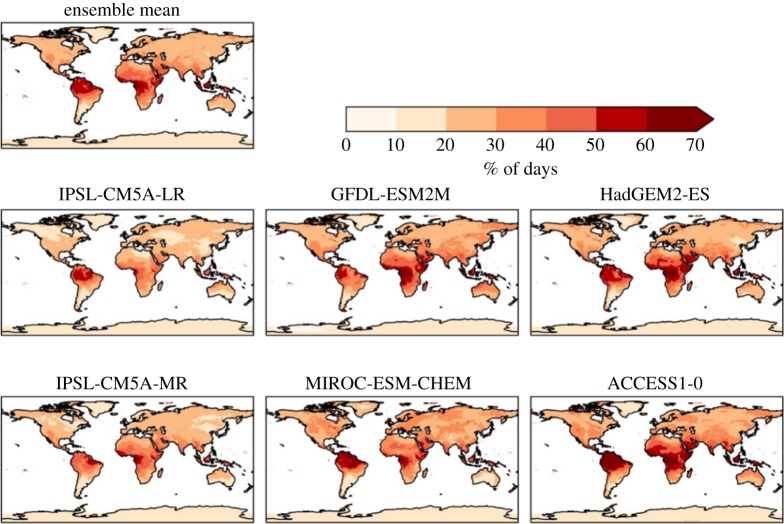
Simulated changes in the percentage of days with daily temperature above the 90th percentile for 1981–2010 at 2°C global warming, for individual HadGEM3 simulations driven by SSTs and SICs from different members of the CMIP5 ensemble, and the ensemble mean. The labels above each panel identify the driving CMIP5 model (or ensemble mean).

The percentage of days exceeding the 90th percentile of daily maximum temperature increase more in tropical areas (figure 3). Some areas show over 60% of days above this level at 2°C global warming compared with present day, whereas in the mid-latitudes between 20% and 30% of days exceed this level. The global mean is between 20% and 30% in all ensemble members (table 3).

Indices based upon daily precipitation often show more spatial variability in changes than the temperature-based indices, and greater differences between ensemble members, but, nevertheless, some consistent pictures still emerge.

**Figure 4.**
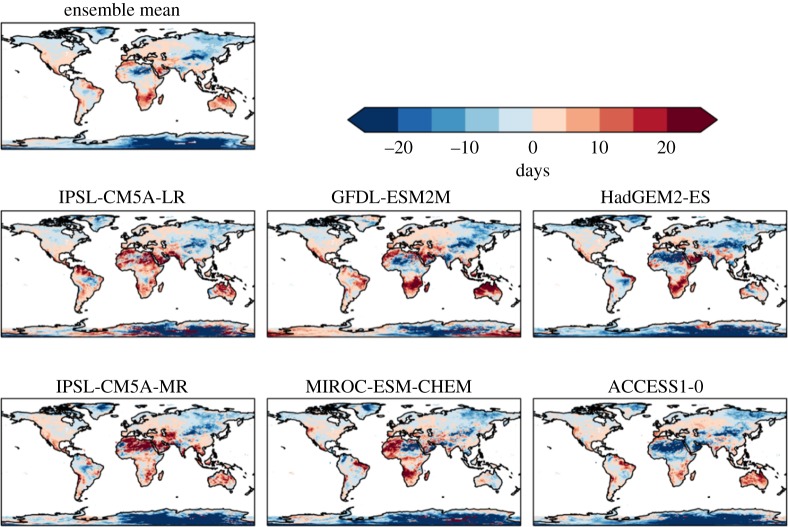
Simulated changes in the number of consecutive dry days relative to 1981–2010, at 2°C global warming, for individual HadGEM3 simulations driven by SSTs and SICs from different members of the CMIP5 ensemble, and the ensemble mean. The labels above each panel identify the driving CMIP5 model (or ensemble mean).

The number of consecutive dry days is projected to increase over some regions and decrease in others (figure 4). Southern Africa, the Mediterranean, Australia and northeast South America are projected to have increased dry spell lengths, while this is projected to decrease in central and eastern Asia. The general pattern of these projections is broadly consistent across the ensemble members. However, the global mean changes vary in sign (table 5), as a result of different magnitudes of regional changes dominating in different ensemble members.

**Figure 5.**
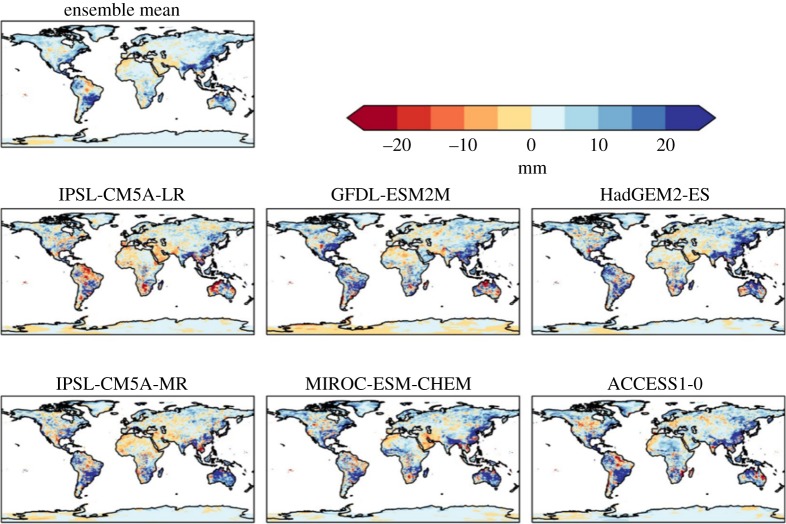
Simulated changes in the annual maximum rainfall over 5 days relative to 1981–2010, at 2°C global warming, for individual HadGEM3 simulations driven by SSTs and SICs from different members of the CMIP5 ensemble, and the ensemble mean. The labels above each panel identify the driving CMIP5 model (or ensemble mean).

Perhaps more surprisingly, projected changes in maximum 5 day rainfall (Rx5day) also vary in sign both geographically and between models (figure 5). Extreme rainfall might simplistically be expected to increase in a warmer climate, and indeed the global mean change is a consistent increase in all ensemble members (table 5). Regional Rx5day is projected to increase over many regions including parts of southeast Asia, southern South America, northern Australia and the east coast of the USA. However, some regions (particularly, the central Amazon and the northern coast of South America) are projected to see a decrease in Rx5day.

Large increases in Rx5day are simulated in south and southeast Asia in all models, but with local details varying. Southeastern South America (broadly southern Brazil and northern Argentina) also see large increases in Rx5day in all models. All models show only small changes over central and north Africa, Europe and most of Asia. In northern South America, however, some models show increases in Rx5day but others show decreases. This suggests that the ensemble-mean result of a decrease in Rx5day in this area may be subject to large uncertainty. Inter-model variations in the sign of changes are seen in a few other local localized regions.

**Figure 6.**
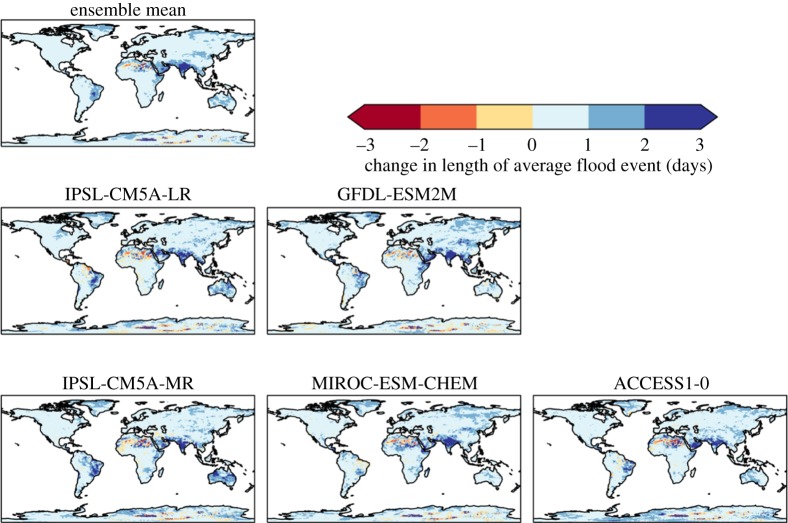
Simulated changes in the average length of flood events (number of days in which the cumulative daily rainfall excess is positive, compared with the 95th percentile in 1981–2010, at 2°C global warming, for individual HadGEM3 simulations driven by SSTs and SICs from different members of the CMIP5 ensemble, and the ensemble mean. The labels above each panel identify the driving CMIP5 model (or ensemble mean).

The average length of flood events (number of days in which the cumulative daily rainfall excess is positive, compared to the 95th percentile of the baseline) generally increase over most of the land surface, although this increase was mostly by a day or less (figure 6). However, some areas are projected to see an increase in flood event lengths of 4 days or more, particularly India and Bangladesh, for which such increases are projected in all ensemble members to some extent. Increases of 2–4 days are also projected in parts of Brazil by all ensemble members, although the magnitude and location within the country varied between members. Similar increases are projected in the region of the Horn of Africa and southern Arabian Peninsula in several members.

**Figure 7.**
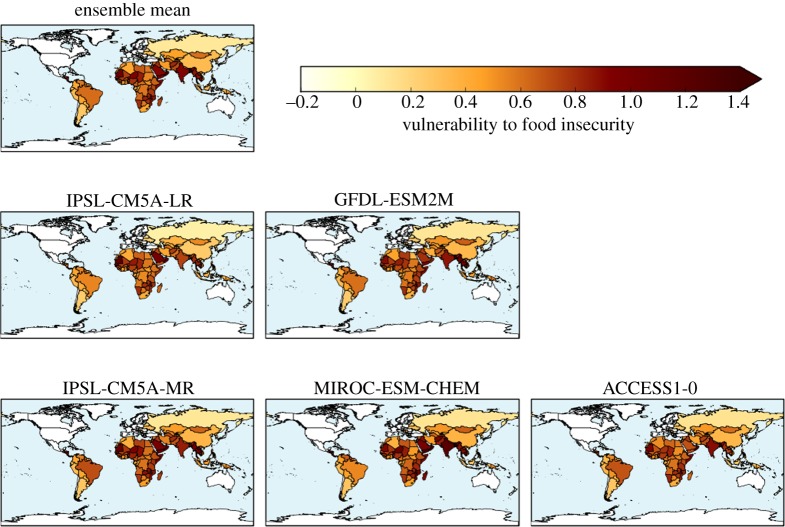
Hunger and Climate Vulnerability Index calculated for simulated climate states at 2°C global warming for five individual HadGEM3 simulations driven by SSTs and SICs from different members of the CMIP5 ensemble, and the ensemble mean.

The HCVI calculated for 2°C global warming showed very large geographical variability (figure 7) which relates largely to differences in socio-economic factors [22]. Differences in the climate change simulated in different ensemble members leads to some variation in the HCVI at 2°C, although the geographical variation is still dominated by the non-climatic factors (figure 7). Therefore, the ensemble-mean change is a reasonable guide to the results.

**Figure 8.**
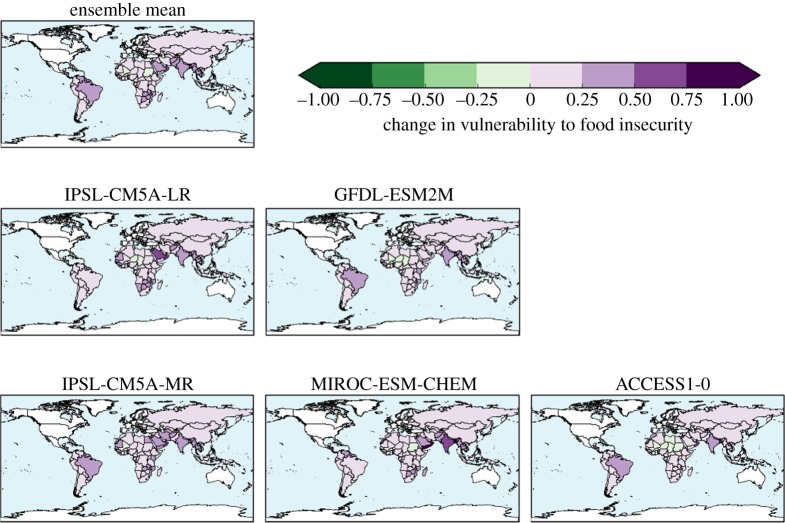
Change in Hunger and Climate Vulnerability Index relative to baseline calculated for simulated climate states at 2°C global warming, for five individual HadGEM3 simulations driven by SSTs and SICs from different members of the CMIP5 ensemble, and the ensemble mean.

The ensemble mean is higher in nearly all assessed countries relative to the baseline (figure 8). The greatest increase was in Oman, followed by India, Bangladesh and Saudi Arabia, then Brazil and a number of its neighbouring countries. Smaller increases in HCVI were seen across Africa. Southeastern Africa showed larger increases than Central Africa. The HCVI decreased in three countries: Mali, Burkino Faso and Sudan.

The ensemble members showed broadly consistent changes in HCVI at 2°C global warming, with increases in most assessed countries and generally similar sets of countries experiencing the largest and smallest changes. Southeastern Africa consistently showed larger increases in HCVI than Central Africa, due to increased length of drought events projected in all ensemble members (not shown). The length of flood events was not projected to increase in this region. The Sahel region consistently showed one or more countries with a small decrease in the HCVI, although the precise country or countries varied between ensemble members. The decrease in HCVI here was due to projected decreases in length of drought, with length of flood events projected to change little.

India is projected to see increased HCVI by all ensemble members, due to a consistent increase in length of flood events projected in all members, outweighing the beneficial impact of decreased length of drought which is again projected in all members.

Brazil is projected to see increased HCVI, but for reasons which vary between ensemble members. Although the location of projected longer flood events varies across the country in different members, the aggregation of the HCVI to the country level renders this geographical variability irrelevant for such a large country because only the median value across the country is used in the HCVI. Some ensemble members project longer drought for Brazil, which again contributed to increased HCVI.

Four countries show ensemble-mean HCVI values at 2°C global warming that are higher than any seen in the baseline climate; these are Oman, Bangladesh, Mauritania and Yemen. The implication of such HCVI values is that climate change at 2°C is projected to cause levels of vulnerability to food insecurity that are greater than any seen in the present day. For individual ensemble members, the number of countries with ‘unprecedented’ HCVI values at 2°C varies from three to seven. Conversely, many countries in the baseline climate have levels of vulnerability to food insecurity that are greater than those expected in other countries under 2°C global warming. This suggests that other factors are already posing greater risk for food insecurity than 2°C climate change is expected to cause in other countries, so the increased risk from climate change should not overshadow the need to reduce vulnerability to food insecurity arising from non-climatic factors. There is scope to reduce vulnerability to food insecurity by addressing various socio-economic issues in such counties.

**Figure 9.**
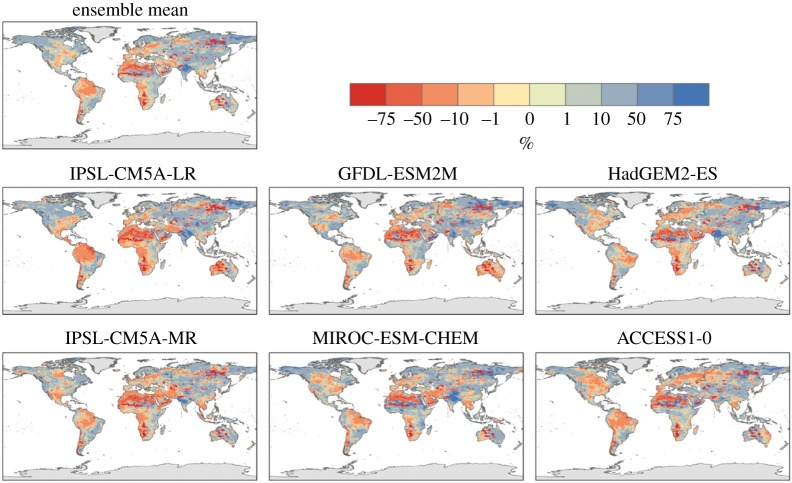
Changes in run-off for mean flows simulated by the JULES ecosystem–hydrology model under six climate simulations at 2°C global warming. (*a*) Ensemble mean and (*b*) percentage of models agreeing on increased flow.

**Figure 10.**
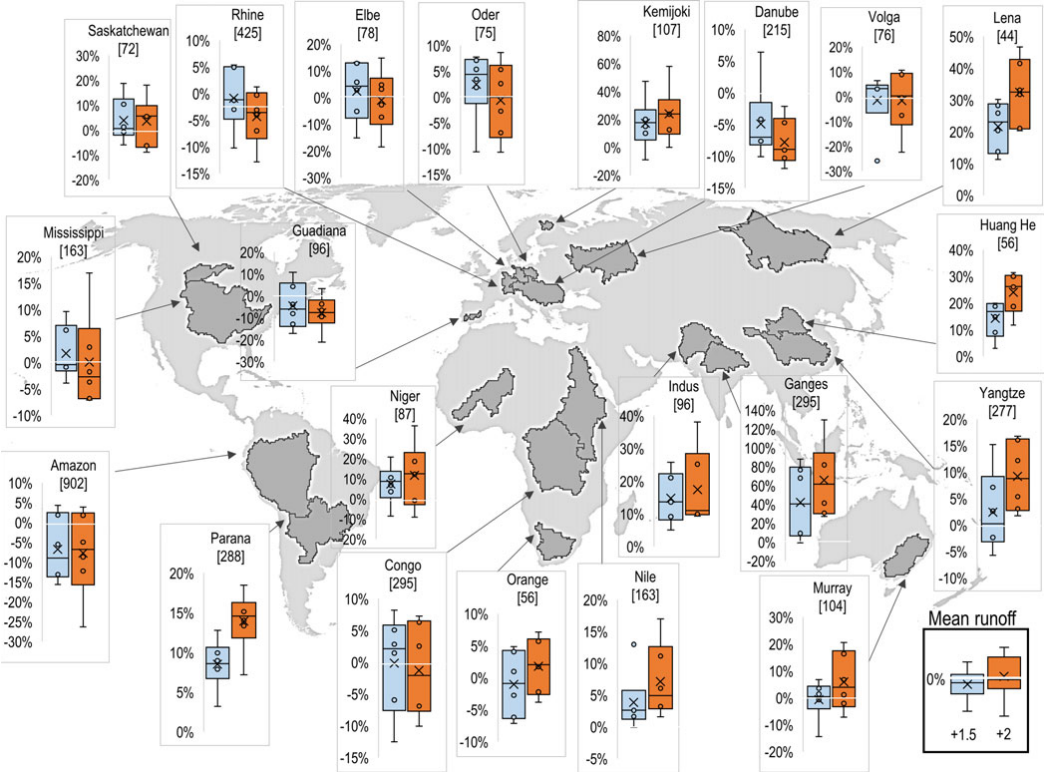
Distributions of changes in run-off for mean flows simulated by the JULES ecosystem–hydrology model under the ensemble of six climate projections at 1.5°C (blue) and 2°C (orange) global warming. Boxes show the 25th and 75th percentile changes, whiskers show the range, circles show the four projections that do not define the ends of the range, and crosses show the ensemble means. Numbers in square brackets show the ensemble-mean flow in the baseline, in millimetres of rain equivalent.

The JULES simulations show a general tendency towards increased run-off over approximately half of the land surface (figure 9) and the majority of the major river basins assessed (figure 10), but with large regional uncertainties including the possibility of decreased flows in many basins. The ensemble-mean change in mean streamflow shows an increase of between 5 and 25% over most of the Northern Hemisphere land surface, with some regions seeing an increase of over 50% at 2°C global warming. Notable exceptions to this are western Europe and southcentral USA, which see less than a 5% change in run-off, and the already very dry region of the Sahara Desert where the existing very small run-off become even smaller.

**Figure 11.**
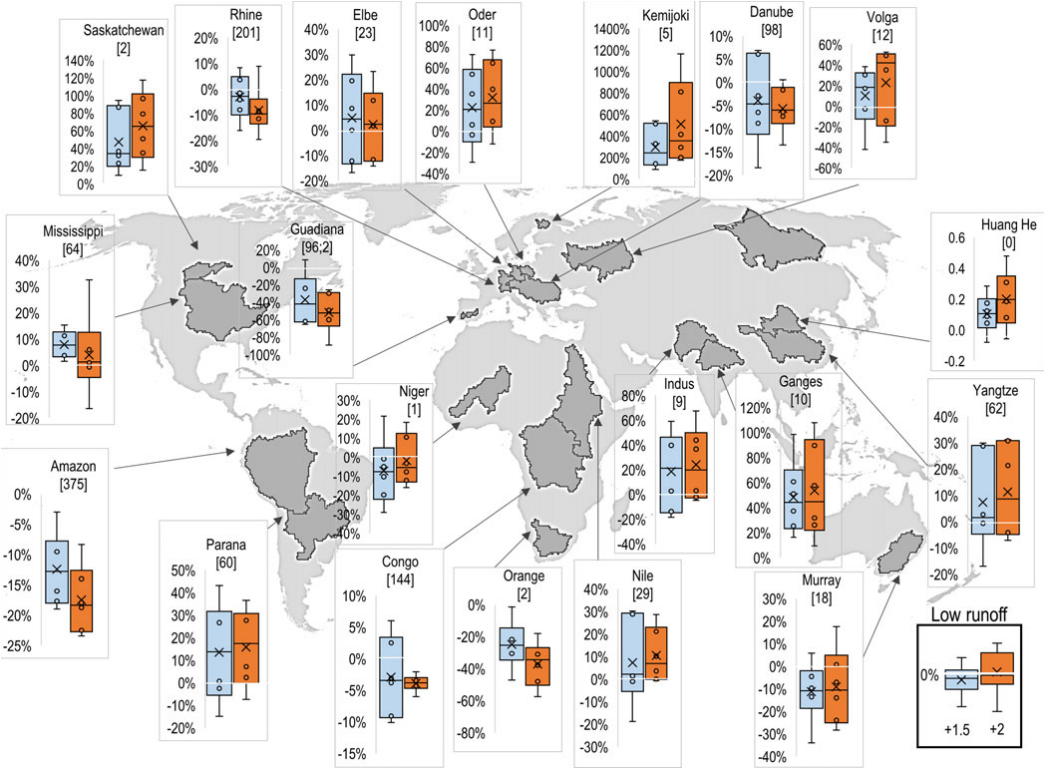
Distributions of changes in run-off for low flows (flows for lowest 10% of time) simulated by the JULES ecosystem–hydrology model under the ensemble of six climate projections at 1.5°C (blue) and 2°C (orange) global warming. Boxes show the 25th and 75th percentile changes, whiskers show the range, circles show the four projections that do not define the ends of the range, and crosses show the ensemble means. Numbers in square brackets show the ensemble-mean flow in the baseline, in millimetres of rain equivalent.

Ensemble-mean projected changes in low run-off flows are generally larger (figure 11), with the regions seeing an increase in mean run-off seeing a larger percentage increase in low run-off—over 75% increases over much of North America, Eastern Europe and Asia. Note that this does not necessarily imply a larger increase in absolute low flow compared to absolute mean flow, because the baseline is (by definition) smaller for low flows. In western Europe, where the changes in mean flows were less than 5%, the ensemble-mean low flow decreases by between 5 and 75%, especially in the Iberian Peninsula. Southern Africa also sees a decrease in low flows where changes in mean flows were small. Changes in high run-off show similar patterns and magnitudes to those in mean run-off.

The simulated changes in both mean and low run-off flows show substantial differences among the six simulations (figures 10 and 11). In most basins examined here, the range of outcomes include both increases and decreases in mean and low flows for any particular basin, but generally with the largest proportion simulating increases in both mean and low flows. In a few cases, notably the Lena in northeast Asia and Ganges in southeast Asia, the ensemble agreed entirely or almost entirely on increased flows. Even here, the range of outcomes is large, with the projected flow increases in the Ganges for 2°C global warming ranging from approximately 30% to more than 110%.

Exceptions to the general picture of consensus on increasing flows are seen in the Amazon, Orange, Danube and Guadiana basins where the range of projected extends more towards decreased mean flows. Mean flows in the Amazon are projected to decline by up to 25% for 2°C global warming. For low flows, the ensemble of projections entirely gives decreased flows at 2°C global warming for these basins.

The signal of decreased flows was stronger for low flows than mean flows, and indeed in the Niger, the range of mean flow changes extended more towards increases whereas the range of low flow changes extended more towards decreases.

### (b) Impacts at 1.5°C global warming compared to 2°C

**Figure 12.**
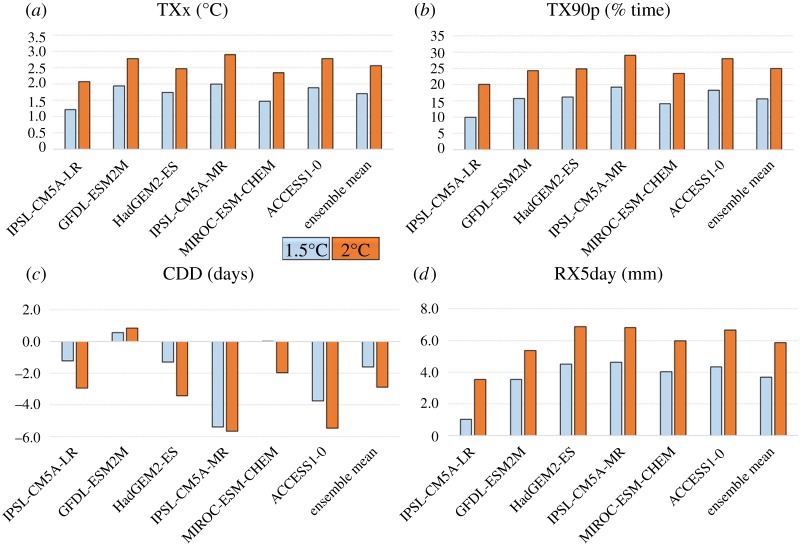
Comparison of global mean changes in climate extremes indices relative to 1981–2010 at 2°C and 1.5°C global warming for individual ensemble members and ensemble mean. (*a*) Change in annual daily maximum temperature; (*b*) percentage of days with maximum temperature above 90th percentile for 1981–2010; (*c*) change in consecutive dry days; (*d*) change in annual maximum 5-day rainfall.

**Figure 13.**
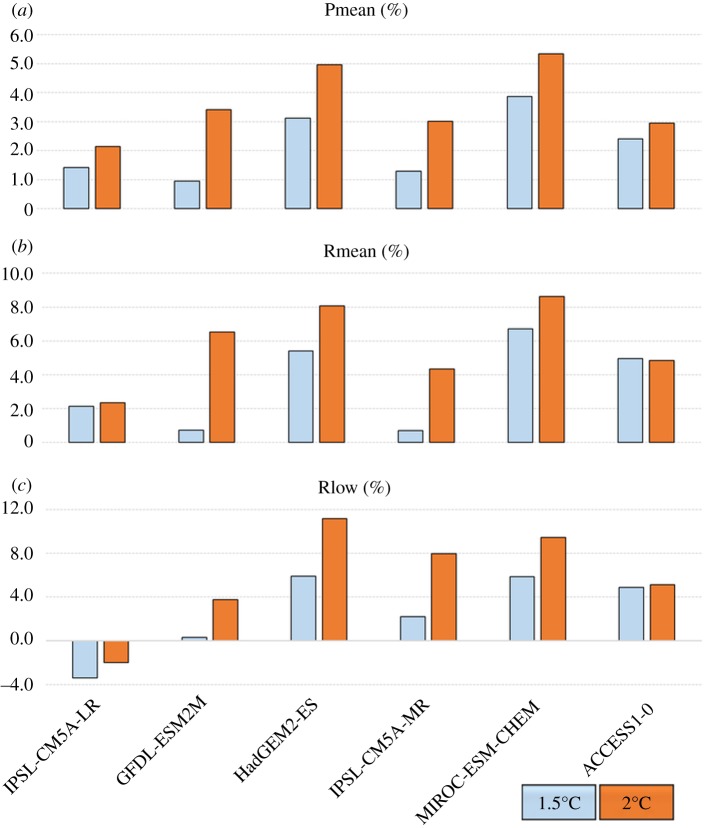
Global mean percentage changes relative to 1981–2010 in (*a*) precipitation over land, (*b*) mean run-off flows, (*c*) low run-off lows (10th percentile), at 2°C and 1.5°C global warming.

For almost all quantities and simulations examined here, global-scale changes in extremes and run-off at 1.5°C global warming (table 6) are smaller than those compared to 2°C (table 5; figures 12 and 13). The exceptions to these are mean and low run-off which each show one instance of a smaller change at 2°C than 1.5°C, but still with a majority of simulations showing larger changes at 2°C (figure 13). For temperature-related indices, the ranges of change at the two GWLs do not overlap—the change at 2°C in all members is larger than the change at 1.5°C in all members (figure 12). This is not the case for the precipitation and run-off results; for those quantities, there is substantial overlap in the ranges of changes at 2°C and 1.5°C, so there is not a consistent picture of how much wetter or drier the world is projected to be in this ensemble, even though it involves a single atmosphere model.

**Table 6.** Global mean changes at 1.5°C global warming compared to present day for individual ensemble members, for the ClimPACT indices, the flood and drought proxies used as input to the HCVI calculations, and percentage change in mean precipitation (Pmean), mean run-off (Rmean) and low run-off (Rlow).

	IPSL-CM5A-LR	GFDL-ESM2M	HadGEM2-ES	IPSL-CM5A-MR	MIROC-ESM-CHEM	ACCESS1-0	ensemble mean
TXx (°C)	1.2	1.9	1.7	2.0	1.5	1.9	1.7
TX90p (% time)	10.0	15.7	16.2	19.2	14.1	18.3	15.6
CDD	−1.2	0.7	−1.3	−5.4	0.0	−3.8	−1.6
RX5day (mm)	1.1	3.6	4.5	4.6	4.0	4.3	3.6
drought proxy	0.74	0.48	n.a.	0.39	0.16	0.31	0.42
flood proxy	0.75	0.73	n.a.	0.73	0.79	0.73	0.75
Pmean (%)	1.4	0.9	3.1	1.3	3.9	2.4	2.2
Rmean (%)	2.1	0.7	5.4	0.7	6.7	5.0	3.9
Rlow (%)	−3.4	0.3	5.9	2.2	5.9	4.9	2.6

**Figure 14.**
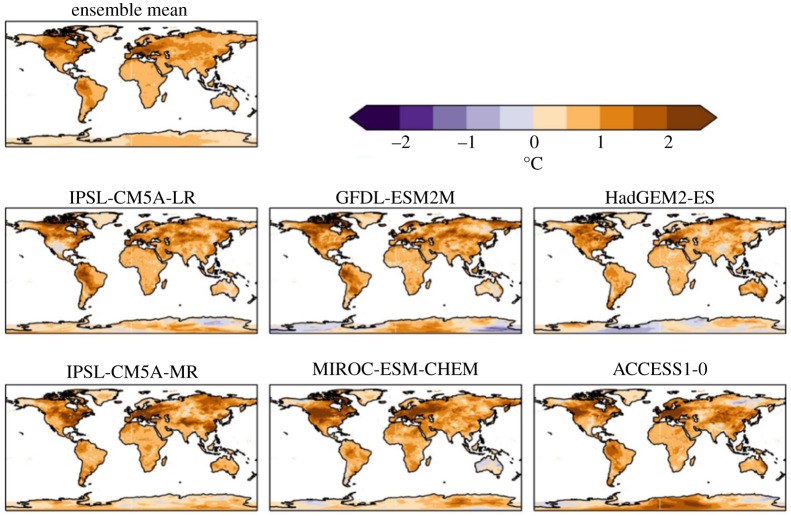
Difference in annual maximum daily maximum temperature between 2°C and 1.5°C global warming, for individual ensemble members and ensemble mean.

For TXx, the difference between 2°C and 1.5°C global warming is larger than the 0.5°C difference in global mean temperature across most of the land surface in all ensemble members (figure 14). Although some ensemble members simulate local temperatures to be higher at 1.5°C global warming than 2°C in some small regions, these are relatively localized and most regions are cooler at 1.5°C global warming than 2°C. In many regions, the difference is between 0.5°C and 1.0°C, but many other regions see larger differences. In several ensemble members, the difference is 1.5°C, 2°C or larger in large parts of North America, South America, Europe and China. For example, over parts of Europe, where annual maximum daily temperature was projected to increase by over 5°C for a 2°C global warming, the local increase is limited to 3–4°C for 1.5°C global warming. Limiting global warming by half a degree Celsius would, therefore, limit maximum temperatures by three or four times as much in those areas (figure 14).

At 1.5°C global warming, although the increases in TXx are smaller than at 2°C, these increases show similar geographical patterns as for 2°C in all ensemble members, with larger changes in continental interiors especially in the mid-latitudes (not shown).

**Figure 15.**
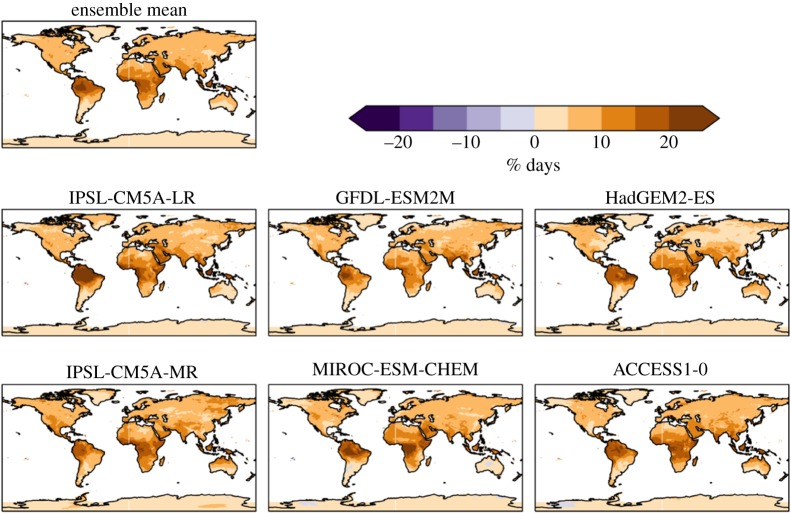
Difference between 2°C and 1.5°C global warming for percentage of days with maximum temperature above 90th percentile of baseline, for individual ensemble members and ensemble mean.

The percentage of days exceeding the 90th percentile of daily temperature (Tx90p) also increases less at 1.5°C global warming than at 2°C (figure 15). The largest reductions are in the tropics, where the largest increase was seen at 2°C; whereas at 2°C global warming, 50% or more days were projected to exceed the baseline 10th percentile, at 1.5°C this reduces by 15–20% or more. Again, the patterns of change at 1.5°C retain a similar geographical pattern of greater increases in the tropics than mid-latitudes (electronic supplementary material).

**Figure 16.**
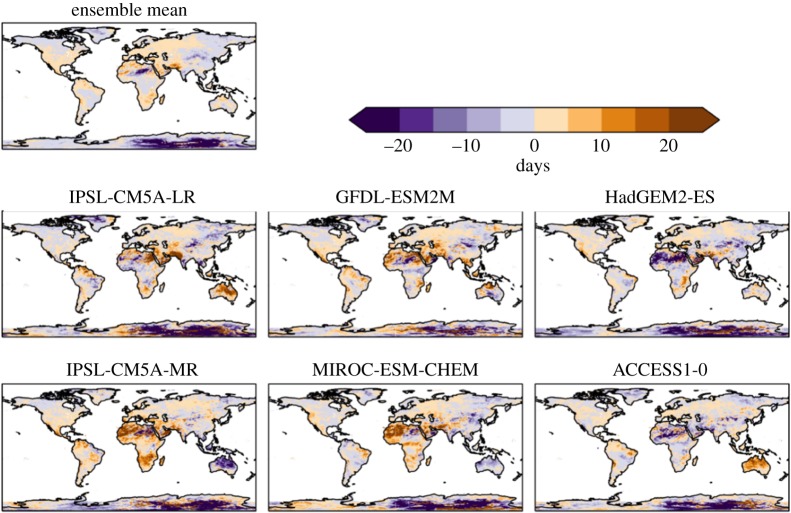
Difference in consecutive dry days between 2°C and 1.5°C global warming, for individual ensemble members and ensemble mean.

For precipitation, generally similar changes are seen at 1.5°C global warming as at 2°C, but smaller in magnitude (compare figures 16 and 4), suggesting that most of these changes are a response to radiatively forced climate change as opposed to internal climate variability. However, some localized changes do vary in sign between the GWLs, such as in South Australia, suggesting a possible dominance of internal variability over the global warming signal in these places.

**Figure 17.**
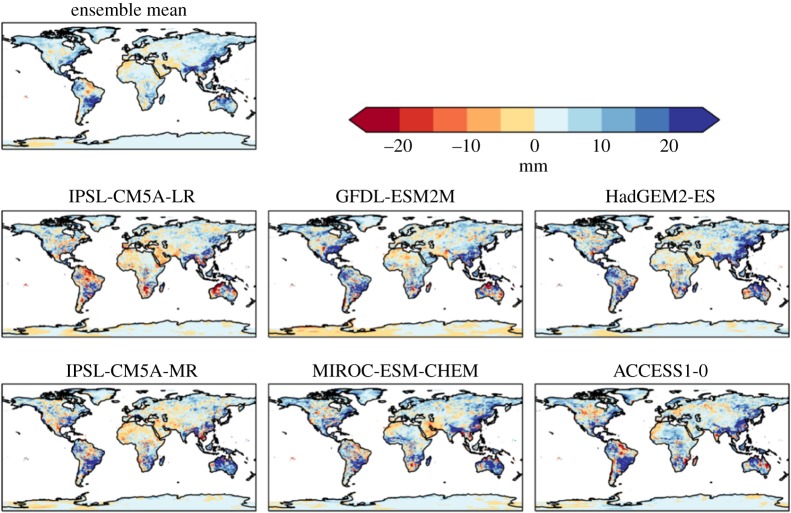
Difference in annual maximum 5 day rainfall between 2°C and 1.5°C global warming, for individual ensemble members and ensemble mean.

Where Rx5day increases, the increases are projected to be larger—in some cases approximately double—at 2°C global warming than 1.5°C. Where Rx5day decreases, again the decreases are projected to be larger at 2°C global warming than 1.5°C (figure 17).

**Figure 18.**
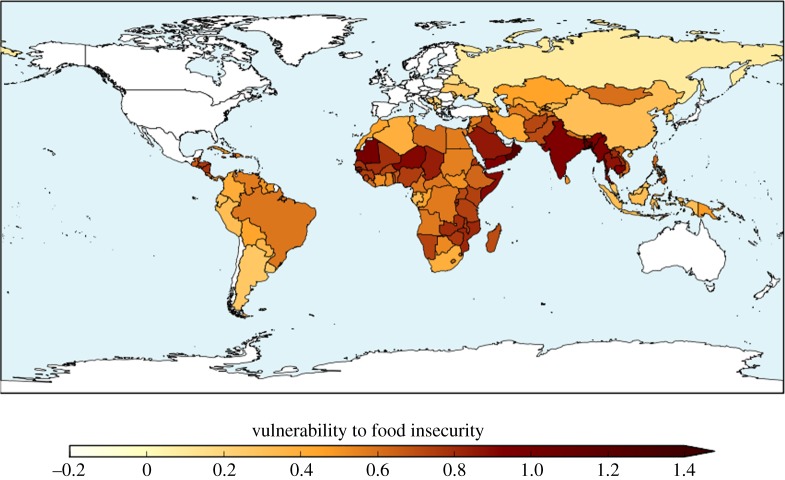
Hunger and Climate Vulnerability Index at 1.5°C global warming (ensemble mean).

**Figure 19.**
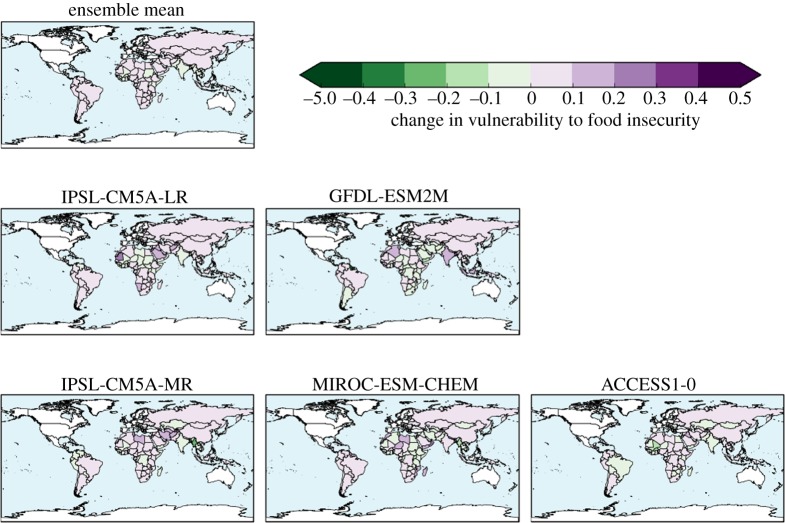
Difference in Hunger and Climate Vulnerability Index between 2°C and 1.5°C global warming, for individual ensemble members and ensemble mean.

Of the 122 countries assessed, 93 have smaller ensemble-mean HCVI calculated at 1.5°C global warming than at 2°C, indicating an ensemble consensus that 76% of assessed countries would see a smaller increase in vulnerability to food insecurity if global warming were limited to 1.5°C (figures 18 and 19). Conversely, 24% of countries would, by this metric, see the same or higher vulnerability to food insecurity at 1.5°C than 2°C. Of these, some are countries where HCVI is projected to be lower at 2°C global warming than in the baseline. For example, in Mali the ensemble-mean baseline HCVI of 0.83 increased slightly to 0.85 at 1.5°C then reduced to 0.81 at 2°C. In some countries, the ensemble-mean HCVI happened to be identical at both warming levels. In Chad, for example, the baseline HCVI of 0.89 increased to 0.91 at both 1.5°C and 2°C.

As noted above, four countries saw ensemble-mean HCVI values at 2°C above any seen in the baseline, and this number increased to seven at 1.5°C. The same four countries with ‘unprecedented’ HCVI values at 2°C also saw ‘unprecedented’ values at 1.5°C; these were Oman, Bangladesh, Mauritania and Yemen. These were joined by Myanmar, India and Cambodia as having ‘unprecedented’ values at 1.5°C. The role of internal climate variability in the HCVI results needs to be assessed, as does the effect of potential nonlinear interactions between the flood and drought metric. Until the reasons behind these country-specific results are understood, this comparison of the number of ‘unprecedented’ HCVI values at 1.5°C and 2°C should be treated with caution. Nevertheless, the finding that some countries see HCVI values higher at either or both 1.5°C and 2°C compared to the baseline may indicate that climate change has the potential to lead to unprecedented levels of vulnerability to food insecurity in some countries. More robustly, it can be concluded that by this metric, overall worldwide vulnerability to food insecurity generally increases with global warming, and for approximately three-quarters of countries assessed, this increase is larger at 2°C than 1.5°C.

**Figure 20.**
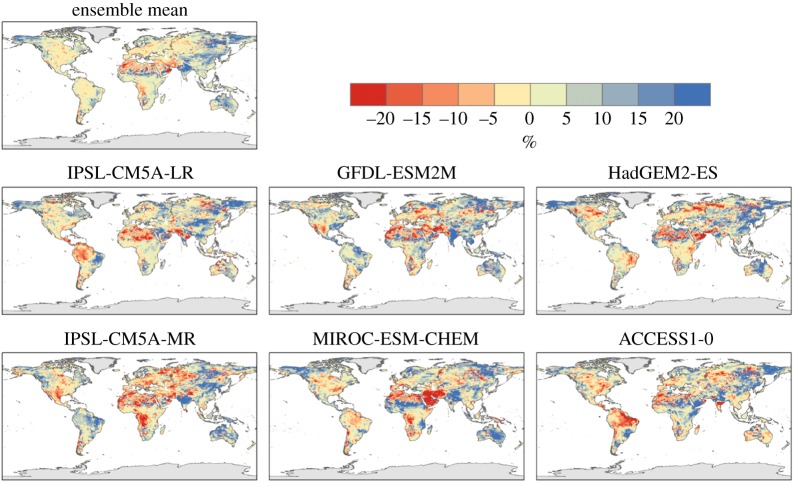
Difference between 2°C and 1.5°C global warming in percentage changes in mean (top) run-off in JULES simulations driven by the ensemble of HadGEM3 simulations. Note that the use of percentage changes emphasizes changes in regions where the baseline streamflow is small.

In the ensemble mean, changes in mean, low and high flows are generally larger at 2°C global warming compared to 1.5°C (figure 20). This is often the case for both increases and decreases in flows—increasing the level of global warming magnifies the pattern of river flow changes, although not in all cases.

The range of projected mean run-off changes is larger for 2°C than 1.5°C in many basins, but this was not always the case, with many basins showing similar or smaller ranges at 2°C compared with 1.5°. Moreover, the ranges overlap substantially, so in terms of the set of possible outcomes projected here, the differences between 2°C and 1.5°C are not always clear. The differences between 2°C and 1.5°C are not always in the same direction as the changes at 2°C; in the Amazon, for example, the difference in flow between 2°C and 1.5°C varies from positive to negative between ensemble members.

## 4. Discussion

In most cases, global mean changes at 2°C are larger than those at 1.5°C, not only for individual members but also for the ensemble as a whole. All ensemble members show increases in TXx at 2°C which are larger than all changes at 1.5°C, and same true for most other variables.

The largest regional differences between 2°C and 1.5°C global warming tend to be in the regions where the local impact is largest relative to the baseline. For TXx this is generally the mid-latitudes, whereas for TX90p it is generally the tropics. So, broadly, the impacts at 1.5°C global warming could be estimated by scaling-back the impacts at 2°C.

These results show some similarities with those from the CMIP5 models [9,38], but also some notable differences. The CMIP5 models were at lower spatial resolution than the models used here. Although the general patterns of change in TXx are broadly similar in our study and CMIP5, with greater warming in many continental interiors, is notable that our results show more marked geographical variation than those from CMIP5 projections ([9], among others), with the continental interior warming being more intense in our projections. In particular, our results with HadGEM3 show more intense increases in maximum temperature in North America and Europe.

Our projections of changes in consecutive dry days (CDD) broadly consistent with those found in a subset of the CMIP5 ensemble [9], although there are some differences. Our ensemble mean suggests shorter dry spells in the central Amazon, whereas ISIMIP-indicated longer dry spells. Also, as with the temperature indices, our results show greater geographical differentiation in the intensity of changes.

The decrease in Rx5day in some regions in our simulations contrasts with the subset of CMIP5 models used for the ISIMIP Fast-Track projections [9] which suggested an increase in Rx5day almost everywhere where at least 66% of the model ensemble agreed on the sign of the change, including all of northern South America. The reasons for these differences require further investigation, but some insight into possible reasons may be gained by examining the similarities and differences between our own individual ensemble members.

For all the CLIMPAct variables, the variations in global means between the ensemble members were consistent at 1.5°C and 2°C. That is, the members with the largest changes at 2°C also showed the largest changes at 1.5°C, and the same was true for the smallest changes, and the relative proportions of changes in other ensemble members. This suggests that variations between the ensemble members at any particular GWL were not merely a consequence of internal variability but also a result of the different forcings influencing the atmosphere model at the time of passing each GWL, and the interaction with the climate sensitivity of HadGEM3. The radiative forcing of non-CO_2_ forcings has previously been highlighted as a potentially important influence on patterns of climate change at 1.5°C and 2°C global warming [39]. Furthermore, despite some differences in regional climate responses between ensemble members, there were also some remarkable consistencies especially in the changes that might be considered inconsistent with a warming climate, such as regions such as northern South America where heavy rainfall (Rx5day) decreases rather increasing as might be expected under a warming climate. Again, these consistencies point to some common forcing of all simulations.

One key factor is the different times of passing a particular GWL, because the net radiative forcing would be different even though the same emissions and concentration scenario was used in all simulations. A given GWL was reached at a different time in each ensemble member, so the CO_2_ and aerosol concentrations vary between ensemble members; in members reaching a GWL early, such as that driven by IPSL-CM5A-LR, the CO_2_ concentration is relatively lower than in other members, and the total aerosol concentration would be relatively higher (CO_2_ concentrations are projected to increase in RCP8.5, but aerosol concentrations are projected decline). The net radiative forcing is smaller, because in RCP8.5 the increase positive radiative forcing from CO_2_ is greater than the decrease in net negative radiative forcing from aerosols. Moreover, the physiological effect of CO_2_ is also smaller, meaning that the consequent reduction in transpiration and associated additional land surface warming influence would also be expected to be smaller.

Conversely, in members reaching the same GWL later, such as that driven by GFDL-ESM2M, CO_2_ concentration is relatively higher, and aerosol concentrations are lower. So, net radiative forcing, CO_2_ physiological effects and the regional-scale radiative forcings from individual aerosol types could, therefore, be quite different in the GFDL-driven HadGEM3 simulation when it reaches 2°C global warming 25 years later than the IPSL-CM5A-LR-driven simulation.

The spatial pattern of changes in the different ensemble members may also play a role in influencing the global mean changes, for example, with large changes in some regions due to faster snow-melt or changes in cloud cover in one ensemble member leading to particular changes in regional warming that are not seen in other ensemble members. Moreover, the individual forcings of the different aerosol components such as sulfate and black carbon differ in sign and spatial pattern, so the overall impact on local radiative forcing and hence regional temperature patterns is more complex. Therefore, the global mean changes may not necessarily be expected to relative to global mean forcings.

A further complexity in identifying precise mechanisms for regional changes is the experimental design used here, with one atmospheric model and concentration/emissions scenario but six different SST and SIC patterns, means that the impact of spatial heterogeneity in radiative forcings is complex and involves a mix of effects in HadGEM3 and the original CMIP5 models. In the case of aerosols, for example, our HadGEM3 simulations are driven with RCP8.5 aerosol emissions and the aerosol concentrations are then calculated within the model itself. The spatial distributions of aerosol optical depth and radiative forcing can, therefore, be expected to be reasonably similar, because they arise from the same emissions scenario, although some differences may occur due to the different regional climate-change patterns. However, the impact of aerosols is also seen in the SST and SIC changes, because these will have responded to changes in regional aerosol radiative forcing in the original CMIP5 simulations. Therefore, these SST and SIC patterns will carry the ‘memory’ of aerosol changes in the original CMIP5 projections.

One example of an impact of changing aerosol radiative forcing could be the precipitation changes in northern South America including Amazonia. All ensemble members show a general drying in this region, as seen in RX5day and mean run-off results. The reduction in Rx5day is particularly notable, because the general expectation would be for an increase in heavy rainfall events in a warmer climate, as is seen in most other regions in these projections. This reduced rainfall in the Amazon region may be associated with the reducing net negative aerosol radiative forcing in the North Atlantic [40]. CO_2_ physiological forcing may also play a role here [41,42].

A detailed investigation of these factors is beyond the scope of this paper; nevertheless, this result illustrates the important point that the nature and patterns of the climate forcing at a particular level of global warming can play an important role in determining the patterns of regional impacts.

## 5. Conclusion

The higher-resolution HadGEM3 simulations project consistent increases in temperature-related extremes, with larger changes at 2°C compared to 1.5°C and local changes being larger than the global annual mean. There is a higher degree of spatial variation in our projections compared with CMIP5-based studies.

In the model projections examined here, changes relating to the water cycle are complex, both in their geographical pattern and in the variation between different models. The length of flooding events generally increases across world in all models, but maximum rainfall can either increase or decrease depending on locations. Global patterns of increase and decrease show some consistency between the different GWLs, but also some local differences. Worldwide, most impacts broadly tend to increase with global warming in most areas. For global mean changes, even when the sign of change is uncertain, individual realizations generally show reduced impact at 1.5°C compared with 2°C. However, this does not always hold even at the scale of major global river basins.

Vulnerability to food insecurity increases more at 2°C global warming than 1.5°C in approximately three-quarters of countries assessed. The vulnerability increase can arise from increases in either flooding or drought. Reduced drought leads to decreased vulnerability in a limited number of cases.

Most simulations here project a general increase in mean streamflow in most of the basins examined, but with a number of notable exceptions in the tropics. While flows in the Ganges are consistently projected to increase by 30–110% at 2°C, Amazon flows could either increase by 3% or decrease by 25%. Ensemble-mean changes in river flow often do not give a full impression of the magnitude of changes that may be possible, so adaptation planning in particular should not rely on ensemble-mean projections and instead consider a range of outcomes. The seasonal low streamflows also increase in many basins, but not as many as for the mean flows—many basins see decreased low flows in some or all projections.

Broadly, changes in weather extremes at 1.5°C global warming could be estimated by scaling-back the impacts at 2°C, if this is done with individual ensemble members rather than the ensemble mean. However, this was not always the case for impacts that depend on more complex process or interactions between more than one climate variable, such as run-off and an indicator of vulnerability to food insecurity.

## Data accessibility

This article has no additional data.

## References

[1] IPCC. 2014 Summary for policymakers. In Climate change 2014: impacts, adaptation, and vulnerability. Part A: global and sectoral aspects. Contribution of Working Group II to the Fifth Assessment Report of the Intergovernmental Panel on Climate Change (eds Field CB *et al*), pp. 1–32. Cambridge, UK: Cambridge University Press.

[2] Murphy JM et al. 2009 UK climate projections science report: climate change projections. Exeter, UK: Met Office Hadley Centre See http://ukclimateprojections.metoffice.gov.uk.

[3] United Nations. 2010 Report of the Conference Parties on its fifteenth session, held in Copenhagen, 7 to 19 December 2009 Addendum. Part Two: Action taken by the Conference of the Parties at its fifteenth session. See http://unfccc.int/resource/docs/2009/cop15/eng/11a01.pdf.

[4] United Nations. 2016 Report of the Conference Parties on its twenty-first session, held in Paris, 30 November to 13 December 2015 Addendum Part two: Action taken by the Conference of the Parties at its twenty-first session. See http://unfccc.int/resource/docs/2015/cop21/eng/10a01.pdf.

[5] Hewitson B et al. 2014 Regional context. In Climate change 2014: impacts, adaptation, and vulnerability. Part B: regional aspects. Contribution of Working Group II to the Fifth assessment report of the Intergovernmental Panel on Climate Change (eds Barros VR et al.), pp. 1133–1197. Cambridge, UK: Cambridge University Press.

[6] Dankers R et al. 2013 First look at changes in flood hazard in the inter-sectoral impact model intercomparison project ensemble. Proc. Natl Acad. Sci. USA 111, 3257–3261. (10.1073/pnas.1302078110) 24344290 PMC3948307

[7] IPCC. 2014 Summary for policymakers. In Climate change 2014: impacts, adaptation, and vulnerability. Part A: global and sectoral aspects. Contribution of Working Group II to the Fifth Assessment Report of the Intergovernmental Panel on Climate Change (eds Field CB et al.), pp. 1–32. Cambridge, UK: Cambridge University Press.

[8] Schewe J et al. 2014 Multimodel assessment of water scarcity under climate change. Proc. Natl Acad. Sci. USA 111, 3245–3250. (10.1073/pnas.1222460110) 24344289 PMC3948304

[9] Schleussner C-F et al. 2015 Differential climate impacts for policy-relevant limits to global warming: the case of 1.5°C and 2°C. Earth Syst. Dynam. Discuss. 6, 2447–2505. (10.5194/esdd-6-2447-2015)

[10] James R, Washington R, Schleussner C-F, Rogeli J, Conway D 2017 Characterizing half-a-degree difference: a review of methods for identifying regional climate responses to global warming targets. WIREs Clim Change 8, e457 (10.1002/wcc.457)

[11] Haarsma RJ et al. 2016 High resolution model intercomparison project (HighResMIP v1.0) for CMIP6. Geosci. Model Dev. 9, 4185–4208. (10.5194/gmd-9-4185-2016) PMC5911933 29697697

[12] Hewitt HT, Copsey D, Culverwell ID, Harris CM, Hill RSR, Keen AB, McLaren AJ, Hunke EC 2011 Design and implementation of the infrastructure of HadGEM3: the next-generation Met Office climate modelling system. Geosci. Model Dev. 4, 223–253. (10.5194/gmd-4-223-2011).

[13] Martin GM et al. 2011 The HadGEM2 family of met office unified model climate configurations. Geosci. Model Dev. 4, 723–757. (10.5194/gmd-4-723-2011)

[14] Walters DN et al. 2011 The Met Office Unified Model Global Atmosphere 3.0/3.1 and JULES global land 3.0/3.1 configurations. Geosci. Model Dev. 4, 919–941. (10.5194/gmd-4-919-2011)

[15] Williams KD et al. 2015 The Met Office Global Coupled Model 2.0 (GC2) configuration. Geosci. Model Dev. 8, 1509–1524. (10.5194/gmd-8-1509-2015)

[16] Senior CA et al. 2016 Idealized climate change simulations with a high-resolution physical model: HadGEM3-GC2. J. Adv. Model. Earth Syst. 8, 813–830. (10.1002/2015MS000614)

[17] Wood N et al. 2014 An inherently mass-conserving semi-implicit semi-Lagrangian discretization of the deep-atmosphere global non-hydrostatic equations. Q. J. R. Meteorol. Soc. 140, 1505–1520. (10.1002/qj.2235)

[18] MacLachlan C et al. 2014 Global seasonal forecast system version 5 (GloSea5): a high-resolution seasonal forecast system. Q. J. R. Meteorol. Soc. 141, 1072–1084. (10.1002/qj.2396)

[19] Knight J et al. 2014 Predictions of climate several years ahead using an improved decadal prediction system. J. Clim. 27, 7550–7567. (10.1175/JCLI-D-14-00069.1)

[20] Wyser K et al. 2016 Documentation of changes in climate variability and extremes simulated by the HELIX AGCMs at the 3 SWLs and comparison to changes in equivalent SST/SIC low-resolution CMIP5 projections. HELIX project deliverable 3.1.

[21] Alexander L, Yang H, Perkins S. 2018 ClimPACT—Indices and Software. User Manual. See http://www.wmo.int/pages/prog/wcp/ccl/opace/opace4/meetings/documents/ETCRSCI_software_documentation_v2a.doc (accessed on 5 February 2018).

[22] Krishnamurthy PK, Lewis K, Choularton RJ 2014 A methodological framework for rapidly assessing the impacts of climate risk on national-level food security through a vulnerability index. Glob. Environ. Change 25, 121–132. (10.1016/j.gloenvcha.2013.11.004)

[23] Richardson K, Lewis K, Krishnamurthy K, Kent C, Wiltshire A, Hanlon H 2018 Food security outcomes under a changing climate: impacts of mitigation and adaptation on vulnerability to food insecurity. Clim. Change, 147, 327–341. (10.1007/s10584-018-2137-y)

[24] Best M et al. 2011 The joint UK land environment simulator (JULES), model description—part 1: energy and water fluxes. Geosci. Model Dev. 4, 677–699. (10.5194/gmd-4-677-2011)

[25] Clark D et al. 2011 The joint UK land environment simulator (JULES), model description–part 2: carbon fluxes and vegetation dynamics. Geosci. Model Dev. 4, 701–722. (10.5194/gmd-4-701-2011)

[26] Cox PM, Betts RA, Jones CD, Spall SA, Totterdell IJ 2000 Acceleration of global warming due to carbon-cycle feedbacks in a coupled climate model. Nature 408, 184–187. (10.1038/35041539) 11089968

[27] Jones CD et al. 2011 The HadGEM2-ES implementation of CMIP5 centennial simulations. Geosci. Model Dev. 4, 543–570. (10.5194/gmd-4-543-2011)

[28] Betts RA et al. 2015 Climate and land use change impacts on global terrestrial ecosystems and river flows in the HadGEM2-ES Earth system model using the representative concentration pathways. Biogeosciences 12, 1317 (10.5194/bg-12-1317-2015)

[29] Falloon PD, Betts RA 2006 The impact of climate change on global river flow in HadGEM1 simulations. Atmos. Sci. Lett. 7, 62–68. (10.1002/asl.133)

[30] Wiltshire A, Gornall J, Booth B, Dennis E, Falloon P, Kay G, McNeall D, McSweeney C, Betts R 2013 The importance of population, climate change and CO_2_ plant physiological forcing in determining future global water stress. Glob. Environ. Change 23, 1083–1097. (10.1016/j.gloenvcha.2013.06.005)

[31] Papadimitriou LV, Koutroulis AG, Grillakis MG, Tsanis IK 2016 High-end climate change impact on European runoff and low flows – exploring the effects of forcing biases. Hydrol. Earth Syst. Sci. 20, 1785–1808. (10.5194/hess-20-1785)

[32] Milly PCD, Dunne KA 2016 Potential evapotranspiration and continental drying. Nat. Clim. Change 6, 946–949. (10.1038/nclimate3046)

[33] Swann ALS, Hoffman FM, Koven CD, Randerson JT 2016 Plant responses to increasing CO_2_ reduce estimates of climate impacts on drought severity. Proc. Natl Acad. Sci. USA 113, 10 019–10 024. (10.1073/pnas.1604581113) 27573831 PMC5018756

[34] Betts RA et al. 2007 Projected increase in future river runoff through plant responses to carbon dioxide rise. Nature 448, 1037–1042. (10.1038/nature06045) 17728755

[35] Papadimitriou LV, Koutroulis AG, Grillakis MG, Tsanis IK 2017 The effect of GCM biases on global runoff simulations of a land surface model. Hydrol. Earth Syst. Sci. 21, 4379–4401. (10.5194/hess-21-4379-2017)

[36] Sheffield J, Goteti G, Wood EF 2006 Development of a 50-year high-resolution global dataset of meteorological forcings for land surface modeling. J. Climate 19, 3088–3111. (10.1175/JCLI3790.1)

[37] Grillakis MG, Koutroulis AG, Tsanis IK 2013 Multisegment statistical bias correction of daily GCM precipitation output. J. Geophys. Res. Atmos. 118, 3150–3162. (10.1002/jgrd.50323)

[38] Wartenburger R, Hirschi M, Donat MG, Greve P, Pitman AJ, Seneviratne SI 2017 Changes in regional climate extremes as a function of global mean temperature: an interactive plotting framework. Geosci. Model Dev. 10, 3609–3634. (10.5194/gmd-10-3609-2017)

[39] Mitchell D, James R, Forster PM, Betts RA, Shiogama H, Allen M 2016 Realizing the impacts of a 1.5°C warmer world. Nat. Clim. Change 6, 735–737. (10.1038/nclimate3055)

[40] Cox P et al. 2008 Increase risk of Amazonian drought due to decreasing aerosol pollution. Nature 453, 212–216. (10.1038/nature06960) 18464740

[41] Betts RA, Cox PM, Collins M, Harris PP, Huntingford C, Jones CD 2004 The role of ecosystem-atmosphere interactions in simulated Amazonian precipitation decrease and forest dieback under global climate warming. Theor. Appl. Climatol. 78, 157–175. (10.1007/s00704-004-0050-y)

[42] Skinner CB, Poulsen CJ, Chadwick R, Diffenbaugh NS, Fiorella RP 2017 The role of CO2 plant physiological forcing in shaping future daily-scale precipitation. J. Climate 30, 2319–2340. (10.1175/JCLI-D-16-0603.1)

